# Reuse of Heat Resistant Glass Cullet in Cement Composites Subjected to Thermal Load

**DOI:** 10.3390/ma13194434

**Published:** 2020-10-05

**Authors:** Aleksandra Powęzka, Jacek Szulej, Paweł Ogrodnik

**Affiliations:** 1 Faculty of Security Engineering and Civil Protection, Main School of Fire Service, 01-629 Warsaw, Poland; 2 Faculty of Civil Engineering and Architecture, Lublin University of Technology, 20-618 Lublin, Poland; j.szulej@pollub.pl; 3 Institute of Security Engineering, Main School of Fire Service, 01-629 Warsaw, Poland; pogrodnik@sgsp.edu.pl

**Keywords:** recycled aggregates, cullet, cement composite

## Abstract

The article describes the possibility of using waste glass cullet as an alternative aggregate for the production of cement composites. Three concrete mixes based on Portland cement CEM I 42.5 R with different contents of recyclate were designed. Borosilicate glass cullet was introduced into the batch by reducing the content of natural aggregate by 0%, 2.5% and 7.5%. Apparent density, water absorption and compressive strength at elevated temperature were measured. The temperature distribution, in cubic samples, was followed by thermocouples. The elements were heated in a special furnace at the temperatures of 200 °C, 400 °C, 600 °C and 800 °C. The composite topography and phase composition were observed using X-ray energy scattering electron microscopy. The results show that the appropriate modification of the cement composite with 2.5% heat-resistant glass cullet improves both the thermal and mechanical properties. Compressive strength reaches an average value of 48.6 MPa after 28 days. The increase in temperature weakens the structure of the composite. It was found that the obtained cement composite has good physico–chemical properties. The research results are presented in the article.

## 1. Introduction

Glass is commonly used in the construction industry and in various industries branches. It is an inspiration in the search for new applications that require an interdisciplinary approach to the design and implementation processes [1].

Glass is distinguished by its transparency and is also a relatively hard material. Depending on its brand, it occupies the fifth or sixth position in the 10-level Mosh hardness scale. Its main weakness is its fragility. It is made of glass sand (source of silica SiO_2_), borax (source of glass forming component B_2_O_3_), sodium–potassium feldspar (source of Al_2_O_3_), sodium carbonate, limestone and others. Components of glass brands used in the construction sector comprise circa 70–72% SiO_2_, circa 15% Na_2_O, circa 10% CaO, as well as MgO, Al_2_O_3_ and other ingredients adopted to improve the properties of glass or to streamline the production process [2]. Glass is a fragile material, which means that once it reaches its maximum strength, it cracks and becomes crushed. Compared to steel or concrete, glass is characterised by high compressive strength equalling to 0.8–1 GPa. The tensile and flexural strength of glass is much lower and equals 30–90 MPa. In addition, glass has a high Young’s modulus equalling to circa 70 GPa. This value is comparable to the Young’s modulus of aluminium [1]. Apart from traditional functions, glass is meant among others to insulate and to protect from burglary or from a fire. The resistance of ordinary glass to high temperatures, such as those that occur during a fire, is very low. After a few minutes it becomes completely destroyed. Construction glass (single-layer silica glazing) cracks and falls out of the frame after being heated to 200 °C. Much better properties are offered by reinforced silicate glass, which may crack, yet it does not fall out from the frame that holds it. Borosilicate glass is characterised by a high chemical resistance, withstands high temperatures for a longer time, maintains tightness, but concurrently also permits passage of thermal radiation to the protected zone. It is also characterised by “flexibility” at fire temperature that prevents its cracking [3].

Studies carried out to date indicate that glass waste constitutes 7–10% of all waste deposited on landfills. Recycling of cullet limits the usage of sand, limestone, soda, etc., as well as even water, by 50% with concurrent reduction of energy by circa 30%. This allows lowering the emission of contamination to the atmosphere. Approximately 30% of glass packaging items are processed to cullet. Each obtained tonne of glass cullet allows a saving 800 kg of sand, 250 kg of soda and 180 kg of limestone powder. This shows that the usage of glass from recycling contributes to a reduction in the volume of landfills and widely promotes the circular economy [4,5].

In response to social needs, the necessity of maintaining the market competitiveness and a user friendly natural environment, research implemented to date with respect to elementary features of composites using concrete and ceramic recyclates and cullet go along with the concept of sustainable construction engineering. Recycling of waste glass seems to be an advantageous solution in production of raw materials and manufacturing of secondary products, for example in the sector of civil engineering (recycling of construction aggregate) [6,7,8,9,10].

Glass used for the production of concrete needs to undergo a special processing procedure [5,11]. The whole material may be recycled. It should be borne in mind that glass from broken mirrors, window and car glazing, glasses, wine glasses, heat resistant dishes, light bulbs, ceramic products, porcelain and earthenware articles, bowls, plates, grave candles, medication glass packaging, etc. may not be processed in furnaces designated for cullet obtained from packaging elements [12]. Before it may be added to the concrete mixture, recyclate needs to be tested, because it changes properties of the mixture and hardened cement composite; among other effects it delays hydration and prolongs the initial and final setting time.

Numerous researchers are looking for innovative solutions that would meet performance requirements and determine the inventive nature of the use of waste cullet. Recently conducted studies [13,14,15,16,17,18,19,20,21,22,23,24] enable the determination of the suitability of using waste materials for the production of ceramics, binders, mortar, concrete, plaster, bituminous materials etc. For testing requirements, use is made of various types of cullet, from car glazing [25], soda-lime glass from containers [26,27,28], glass from technological lines of the pharmaceutical industry [29,30,31], cathode ray tubes for TV sets and computer screens [32,33], float glass from window glazing [5], glass from fluorescent lamps [13], glass from heat resistant dishes [34] and mirrors, safety glass, crystal glass, and colourless, green and brown packaging glass [34,35,36,37,38,39] among others.

Cullet is used as a substitute for cement, sand and coarse aggregate in concrete, and in addition may be used for stabilisation of natural soils or for the construction of roads and subgrades [5,40]. The powder fraction of ground glass may also be used in concrete as a pozzolanic material [41]. 

When designing a cement composite, cullet may be introduced into concrete in the form of fibres, dust or grains. The addition of glass consisting of fibres [42,43,44,45,46,47,48,49,50] has an advantageous impact on parameters of concrete. Concrete gains a better strength and is resistant to such environmental factors as humidity, solar radiation, thawing and freezing. 

Replacing cement in the concrete mixture with crushed glass powder [37,38,39] allows the attainment of a composite characterised by better workability and improvement of compressive and flexural strength after a prolonged curing time. Recyclate is characterised by pozzolanic reactivity, which is correlated with average compressive strength [10,51].

When using glass cullet as a substitute for sand [34,35,36,37,52], the type of applied glass needs to be taken into consideration. The application of a 10–30% addition of cullet causes a 10–25% drop compressive strength in resistance compared to the control sample [53]. 

Studies performed until now have proven that it is possible to adopt a solution based on replacement of coarse natural aggregate by waste aggregate. Studies took into consideration glass [54,55,56,57] and ceramic waste [58,59]. From the ecological and economic viewpoint this solution appears to be advantageous, proecological and safe for the environment. It does not require a large amount of work as, for example, in the case of fibres or crushing to a powdered form.

A review of published research [5,11] has shown that no analyses have been implemented yet with respect to recycling of used up heat resistant dishes for the production of concrete. While conducting the research, the authors took into account the feasibility of using glass recyclate as an optional supplementing aggregate for the production of cement composites exposed to thermal loads. Analysis was carried out of selected physical, mechanical and structural properties of the composite.

## 2. Materials and Methods 

### 2.1. Objective and Scope of Testing

The objective of the study was to assess the reuse of heat-resistant glass cullet obtained from damaged dishes. During pilot studies, apart from mechanical properties, also determined was the impact of fire temperatures on properties of the designed composite. The article presents the results of tests and analyses of the microstructure of the contact zone of the material.

### 2.2. Waste Glass Cullet

**Figure 1:**
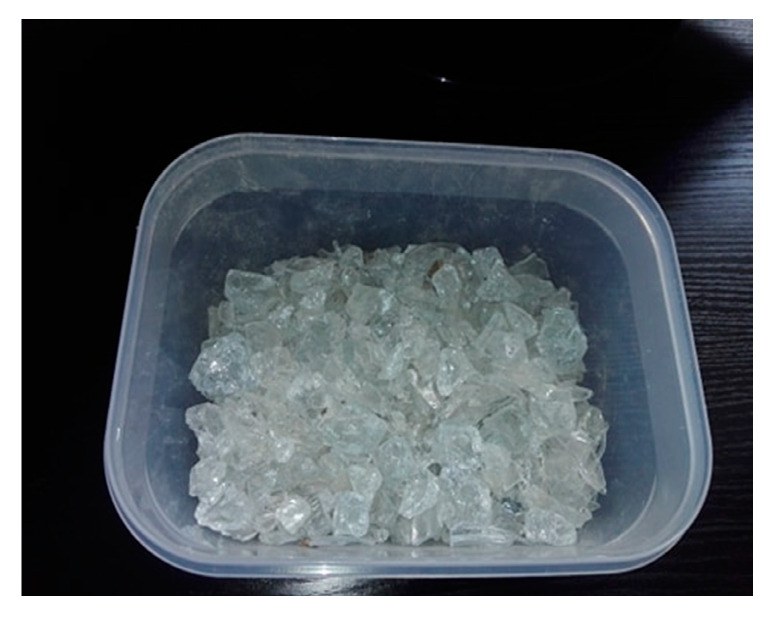
Glass cullet with a fraction of 0/16 mm.

Pilot testing comprised the use of cement composite with diverse additions of recyclate C-0, C-2.5, C-7.5 (C—series of composite made of Portland cement, the number indicates the percentage of glass cullet introduced into the batch by reducing the content of natural aggregate by 0%, 2.5%, 7.5%). A part of the natural aggregate was replaced by borosilicate heatresistant glass cullet. When selecting recycled aggregate it was necessary to take into account first of all advantageous physical parameters and ecological aspects. The material was obtained primarily from broken glass of kitchen and laboratory dishes stocked on premises of the glass works in Wołomin (Termisil S.A. Glassworks, Wołomin, Poland) [5] (Figure 1). Table 1 presents the properties of glass cullet.

Glass ingredients comprise among others silicon oxide (SiO_2_), boron trioxide (B_2_O_3_) or aluminium oxide (Al_2_O_3_). The isotropic material is characterised by higher resistance to thermal shock with a low thermal expansion coefficient. It retains a good thermal resistance in the working temperature range of −40 °C to 500 °C and withstands temperature difference in the range of 150–210 °C. A granulometric analysis of heat resistant glass cullet is presented in Table 2. The content of dust (total from rinsing and sieving) amounts to 16.1 g, while the density of cullet equals 2.25 g/cm^3^ (at 20 °C).

### 2.3. Natural Aggregate

To prepare cement composites quartz sand was used with a fraction of 0/2 mm (PolBot Kruszywa S.A., Warszawa, Poland) and gravel with a grading of 2/16 mm (Zakład Produkcji Kruszyw Szumno Sp. j., Szumowo, Poland).

### 2.4. Cement

All series of samples were produced based on Portland cement CEM I 42.5 R (Cement Ożarów S.A., Ożarów, Poland) and fly ash. The declared physical, chemical and mechanical properties of cement are presented in Table 3 and Table 4.

### 2.5. Fly Ash

Mineral additive was used in the mixture that consisted of fly ash ProAsh cat. A (Zakład Separacji Popiołów Siekierki Sp. z o.o., Warszawa, Poland) with pozzolanic properties. The fly ash fulfils criteria imposed by standard EN 450-1:2012 [60]. Concrete that contains fly ash (amounting to 20%) has better strength and resistance to cracking, corrosion and high temperature [10]. The chemical composition of fly ash is presented in Table 5. Fly ash was used, because composites on a cement base with a 20% additive of ash offer better strength, cracking, corrosion and temperature resistance [5].

### 2.6. Admixtures for Concrete

Concrete mixtures were produced with the use of a plasticiser admixture that reduces the amount of water—Master Pozzolith 18 BVC (BASF Polska Sp. z o.o., Śrem, Poland). The concrete admixture belongs to a group of surfactants based on lignin sulphonates that are known to enhance the moisturising of cement grains by batched water and to assure better workability of the mixture. Physical and chemical properties of the admixture are presented in Table 6.

### 2.7. Water

Potable water was used for production of the concrete mixture.

### 2.8. Preparation of Concrete Mixture

Samples for testing were chosen in a random way. The composition of the source mixture is presented in Table 7.

For all the samples a constant water–cement ratio was assumed that equals 0.62. All mixtures were mixed and compacted thanks to the used chemical admixture 18 BVC (BASF Polska Sp. z o.o., Śrem, Poland) that consists of a superplasticiser based on lignin sulphonates. 

In addition, the aggregate composition and sand content (SC) of concrete mixtures were determined. The sand content of C-0, C-2.5 and C-7.5 amounted to 47%, 49%, 51%, respectively, in conformity with recommendations presented in EN 933-1: 2012 [61]. The samples were produced as cubes with an edge size of 100 mm, which were then stored in laboratory conditions according to requirements of the standard EN 12390-3: 2009 [62]. A part of the natural aggregate in the mixture was substituted by glass cullet. The following series of mixtures were produced: control mixture C-0 containing entirely sand and gravel, C-2.5 containing 2.5% of glass cullet and C-7.5 containing 7.5% of glass cullet (percentage change of aggregate to cullet).

## 3. Research Methods

The technical properties of concrete are a set of all the physical, mechanical, rheological properties and their resistance to the impact of the environment. The article presents a few selected properties of the composite: absorbability, apparent density, compressive strength, deformability under the impact of temperature change (rheological properties), resistance to high temperature and microscopic structure testing.

### 3.1. Granulometric Distribution

Optimum grading is found in the aggregate with the smallest number of cavities between grains, with containing possibly the coarsest aggregate grains. To determine the grading of glass cullet a sieve analysis was carried out. The recyclate was sifted through a set of sieves on a shaker, and then fractions remaining on the sieves and on the bottom were weighed. The test was performed pursuant to standard EN 933-1:2012 [61].

### 3.2. Resistance to Grinding Recyclate

The resistance to grinding was determined by measuring the fragmentation of the aggregate as a result of rolling together the aggregate with steel balls in a rotating drum. The Los Angeles (LA) factor corresponds to the mass of the analytical sample in fragmentation, expressed in %, which has passed through a 1.6 mm sieve after a complete drum rotation cycle. The test was carried out in accordance with the standard PN-EN 1097-2.

### 3.3. Properties of Fresh Concrete

Tests were conducted of the consistence of concrete mixtures according to the standard EN 12350-2:2009 [63]. The mixture was found to have good workability. The achieved consistence class was S2/S3/S4 according to fall of the Abrams cone; the difference in height of the form and receded mixture was in the range of 75–170 mm. The concrete mixture density was tested according to the standard EN 12350-6:2009 [64].

Before the commencement of proper tests, all the samples were weighed and dried to constant mass in the thermal chamber KC-100/200 (Zalmed, Warszawa, Poland), which was repeated until two consequent measurements were found not to differ one from the other by more than 0.1%, at the temperature of 105 °C ± 5 °C. All measurements of the mass were implemented to within an accuracy of 0.001 g. 

### 3.4. Properties of Hardened Concrete 

Test trials were conducted with a view to the determination of physical and mechanical properties. An absorbability test was implemented pursuant to the standard EN 206:2013 [65]. Construction standards define the admissible absorbability of concrete up to 4%. Cement grout coats the grains and hinders the permeation of water, while voids are filled with water and serve as “buffers”. 

Mechanical tests were carried out in laboratory conditions at room temperature with the use of a hydraulic press. The production and curing of concrete samples designated for compressive strength tests were performed according to the standard PN-EN 12390-2 [66]. The samples were cubic formed, with dimensions of 10 cm × 10 cm × 10 cm, and were placed in a climatic chamber. The elements were unmoulded after 24 h, and marked in a durable and legible way by a symbol defining the type of mixture and number of sample. Immediately after the removal of the elements from the moulds, all of them were once again placed on the grates over the water surface in a climatic chamber to mature for 28 days. 

The measurement unit was a Controls Advantest 9 press (Controls, Cernusco sul Naviglio, Italy) including specialist software (Version 2.16-02/2010). Compressive strength is strictly correlated with the microstructure of hardened cement grout and the strength of aggregate and the aggregate–grout contact zone. Compressive strength (f_c_) was determined according to the standard PN-EN 12390-3 [62]. On the basis of obtained average strength values, the result was converted onto cubic samples with side length of 15 cm (according to formula 1) and classes of concrete were defined.

(1) $$f_{c,\;cub\;150}=0.95\times f_{c,\;cub\;100}$$

where: *f_c,cub150_* and *f_c,cub100_*—compressive strength ascertained on cubic samples with edge lengths of 15 and 10 cm, respectively.

### 3.5. Tests of Concrete at Elevated Temperature 

The samples were heated at the temperature of 200 °C, 400 °C, 600 °C and 800 °C in a special furnace PK 1100/5 (Termolab S.C., Warszawa, Poland) furnished with powered heating compartments. The stand has a dedicated programme ThermoPro that allows the programming of the thermal process. The temperature distribution in the analysed element was monitored using NiCr-Ni thermocouples that meet criteria defined by the standard [67].

### 3.6. Scanning Electron Microscopy with Energy Dispersion Microscopy (SEM/EDS)

In order to identify phases based on a qualitative chemical analysis and crystalline structure, to obtain high resolution images of the forms of very small items and to obtain spatial differences in the chemical composition, scanning electron microscopy (SEM) was used. The observations were implemented using the FEG Quanta 250 microscope (FEI, Hilsboro, OR, USA) equipped with an EDS system (EDAX, Mahwah, NJ, USA), based on energy dispersion microscopy used to conduct analyses of the chemical composition with the aim of obtaining maps of the distribution of elements and spot chemical analyses of the material. This enabled the determination of semi-quantitative chemical composition of the observed phases. The EDS detector analyses X-ray radiation that indicates the elemental composition of the tested sample.

Using the X-ray diffractometer (PANalytical, Almelo, The Netherlands), analyses were performed of the topography and phase composition of the designed composite. Materials needed for testing were obtained from fragments of crushed samples remaining from the strength testing.

Samples for SEM testing were coated with powder and fixed to the grip with the use of a carbon adhesive. Next the samples were given a circa 50 nm carbon coating as a result of cathode spraying to assure conductivity on the sample surface.

## 4. Results and Discussion

The objective of the test was to find how waste cullet affects the properties of cement composite. The research population comprised three modified composites (C-0, C-2.5, C-7.5) containing various amounts of recycled aggregate (0%, 2.5%, 7.5%). From each population 15 samples were collected. The experiment includes observations and assessment of selected properties of the sample (statistical features). 

A granulometric analysis was carried out for glass cullet. Tests comprised assessment of the density, absorbability (humidity), compressive strength, resistance to elevated temperature, composite structure, and topography of the tested material. Chemical elements contained in the composites were identified. 

### 4.1. Granulometric Analysis of Waste Cullet using the Sieve Method 

**Figure 2:**
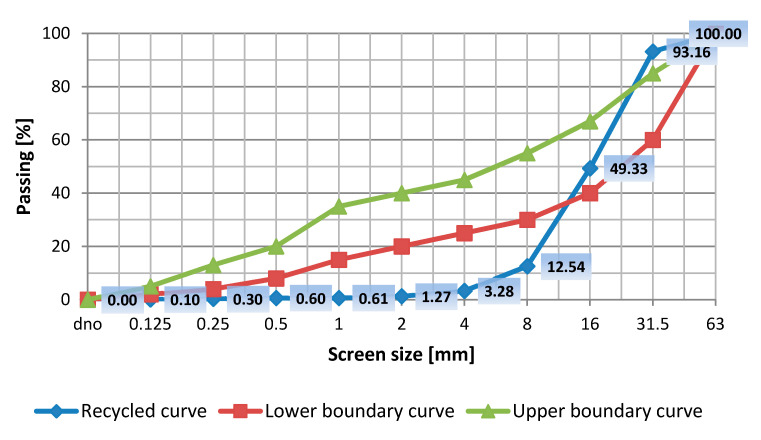
Graphical presentation of the sieve analysis. Grading curves. Recyclate curve compared to the standard good grading field.

Granulometric determination was done on a glass sample obtained from recycling, and then a calculation was made of the degree of uniformity. Results of the sieve analysis were applied onto a semi-logarithmic grid and in such a way that a continuous grading curve was obtained (Figure 2) for recycled aggregate.

The glass aggregate curve is not contained within the area of the limiting sifting curves (upper and lower ones). The sand content of recyclate equals 1.27%. The recyclate fails to meet standard requirements of particularly good grading. The recyclate curve is below the lower limiting curve, yet that does not discriminate recyclate as an additive to concrete. The glass mixture comprises aggregate with a fraction of 0/16 mm.

### 4.2. Aggregate Resistance to Grinding—Los Angeles Method

**Figure 3:**
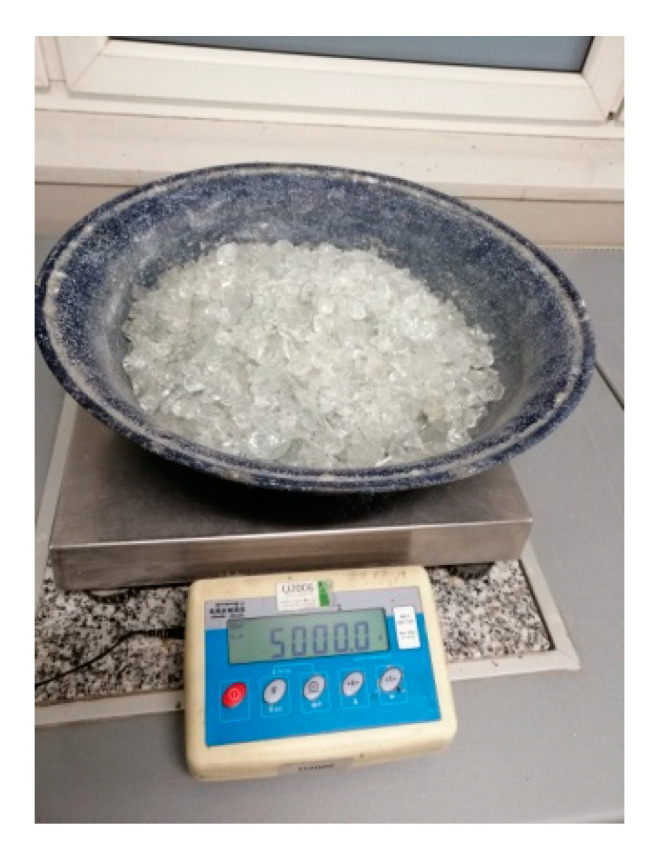
Glass cullet. Analytical sample.

Steel spheres and a 5000 g analytical sample were placed in the Los Angeles drum (Figure 3). 500 rotations of the drum were performed, with a constant speed of 31−33 rpm. After 16 rotation cycles, the sample was wet sieved on a 1.6 mm sieve, according to PN-EN 933–1. The residue on a 1.6 mm sieve was dried at a temperature of 110 ± 5 °C to a constant weight—3134 g. The test result is a Los Angeles (LA) coefficient of 37.32 so the LA fragmentation category is ${\mathrm{LA}}_{40}$. On this basis, the strength class of the cement composite (C16/20 to C30/37) was determined for which the glass cullet can be used.

### 4.3. Physical Properties of Hardened Concrete 

**Figure 4:**
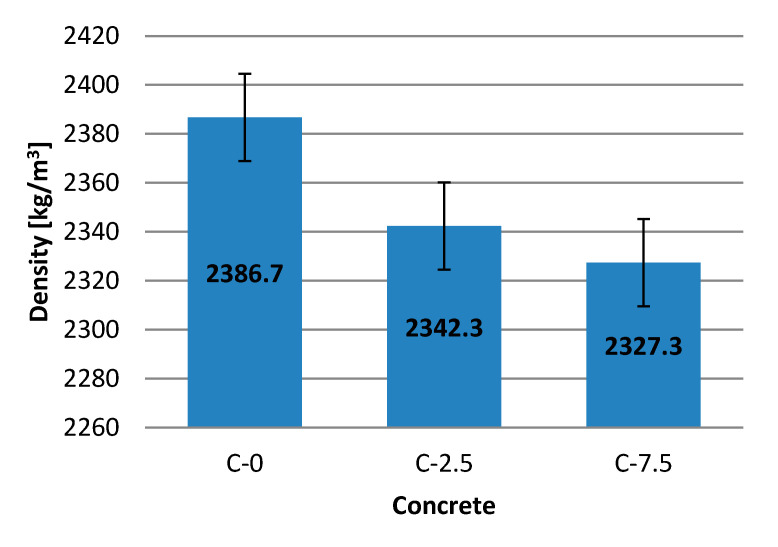
Results of bulk density testing.

Bulk density (apparent density) was tested on cubic samples (100 × 100 × 100) mm. For the testing three samples of each concrete were used. Results of the test are shown in Table 8 and Figure 4. 

Bulk density decreases with the increase in the amount of recyclate in the sample, and differs for all types of concrete. Cement composite C-7.5 with the addition of glass recyclate obtained a density of 2327.3 kg/m^3^. The reduced density could have been caused by building up of the contact zone between the binder and aggregate. The porosity of the composite is also affected by reaction of the used aggregate and the binder. Changes in the zone of adhesive-aggregate were also observed in studies [5,68]. However, in publication [13] certain differences have been observed in density, caused by the growing contents of air in the mixture. 

The subsequent test comprised the determination of total absorbability (humidity) of the material by the weight method. Tests comprised 15 samples of each type of concrete. Next the samples were dried to constant mass. Constant mass is achieved when sample masses does not differ one from another by 0.1%. After saturation with water the samples were heated up to a temperature of 105 ± 5 °C for 72 h, and then they were taken out and weighed. Reinserted for 24 h. After that time, the samples were taken out and measured. A total of seven cycles of this type were carried out.

The saturation time of samples and their drying are determined by standard EN 13369:2018 [69] concerning concrete precast elements. The minimum time amounts to 3 days at change of mass below 0.1%. The authors observed similar drying times of concrete cubes (10 cm) as observed in the studies of [70]. The drying time of samples to constant mass was 6 to 8 days.

**Figure 5:**
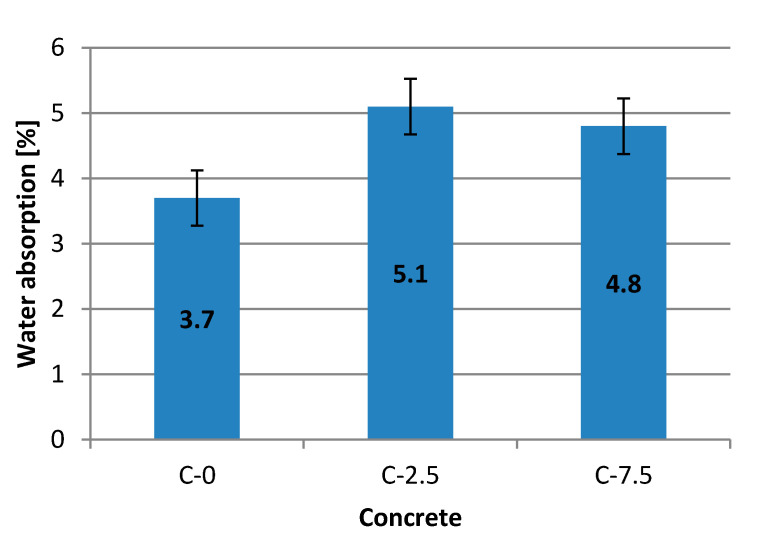
Water absorption of 10 cm cubes.

Figure 5 presents results and an image of changes to absolute humidity of concrete during the process of moisture removal. The elementary descriptive statistics are provided in Table 9.

Water absorption varied, and for mixtures C-0, C-2.5 and C-7.5 amounted to 3.7%, 5.1% and 4.8%, respectively. The difference in results of absorbability was 1.1–1.4%. This proved that, as the amount of glass aggregate grows, the absorption of concrete also grew, most likely also leading to the formation of air voids in the concrete volume. While analysing the results of their own studies, the authors of [5] also drew attention to the increase in the variability index with contents of glass waste. This is further proof that as the amount of waste grows, the samples become less homogeneous.

The figure also shows error bars with standard error that equals 0.05% for concrete C-0 and 0.07% for C-2.5 and 0.06% for C-7.5. The water absorption obtained during testing is lower than 6%. According to standards pertaining to precast concrete and sidewalk elements (pavement, concrete kerbs), they should be considered as satisfactory for any class.

### 4.4. Compressive Strength

**Figure 6:**
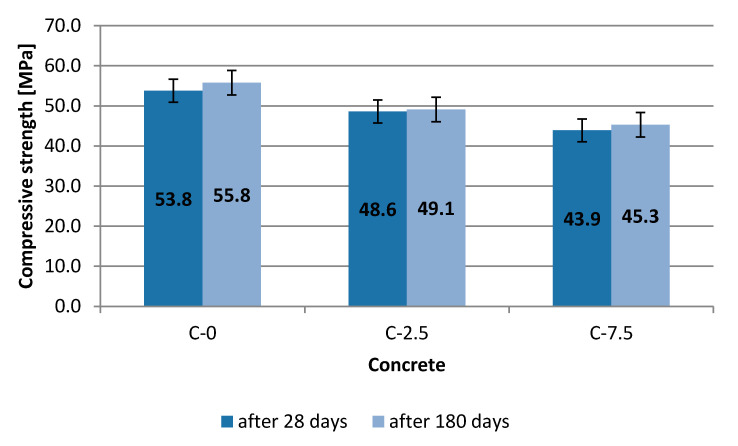
Compressive strength of concrete—10 cm cubes.

The compressive strength test was performed after 28 and 180 curing days of the cement composite. Samples were tested at the laboratory temperature of 20 °C. Three samples were used to determine average strength. The obtained results are presented in the bar chart (Figure 6).

Destructive testing was carried out on samples that had not been subjected to heating. Lower values for concrete C-2.5 and C-7.5 were observed compared to reference concrete C-0 after 28 days of curing. The differences in values vary and appear to have a downtrend. Compressive strength falls with the amount of waste glass in the sample. It has been found that strength after 180 days for C-7.5 was by 3.8 MPa higher than in sample C-2.5 and concurrently lower by 10.5 MPa than for reference concrete C-0. The target strength on control samples amounted to 55.8 MPa. The conducted testing allows the presumption that a higher strength of the composite was obtained by substituting 2.5% of gravel with recyclate. Results obtained from all the tested composites may be considered to be satisfactory. The concrete was classified as C30/37, i.e., lower by one class (C35/45) or two classes (C40/50) obtained after 28 and 180 days by reference concrete C-0.

Table 10 specifies selected statistical parameters for analysed results of compressive strength. Those parameters characterise the homogenous nature of concrete.

**Figure 7:**
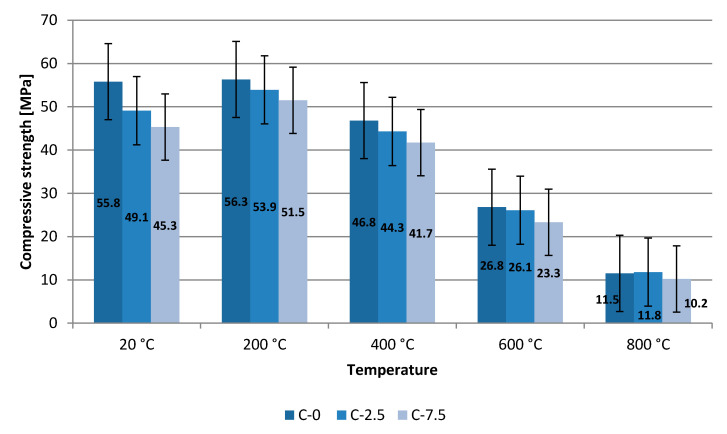
Compressive strength of concrete with heated composite after 180 days—10 cm cubes.

The next destructive testing was performed on heated cubes after 180 days of curing. Heating was performed for three cubes each for temperatures of 200 °C, 400 °C, 600 °C and 800 °C. The heating process progressed in line with the standard temperature-time curve according to EN 1991-1-2. Temperature in the furnace was kept up for a further 60 min until the heating process was completed. Testing of average compressive strength took place at room temperature after total cooling down. Figure 7 shows the results of average strength of concrete samples and presents error bars with the standard error.

An increase was observed in average compressive strength after heating the up of the samples to a temperature of 200 °C. The strength of C-2.5 concrete grew by 4.8 MPa, and for the C-7.5 concrete by 6.2 MPa in relation to the strength determined at room temperature. On the other hand, it may be considered surprising that the strength of composite C-0 increased slightly. The cement composite did not lose its properties thanks to the low coefficient of thermal expansion and the good ability of borosilicate glass to work at temperatures up to 300 °C. The impact of fire temperatures exceeding 400 °C causes a fall in strength by circa 16% (C-0), 10% (C-2.5), 8% (C-7.5). At a temperature of 600 °C the concretes were found to have a reduction of as much as by 50% compared to samples made of reference concrete.

Using smaller contents of recyclate in the concrete has a positive effect on the strength of samples subjected to a fire.

**Figure 8:**
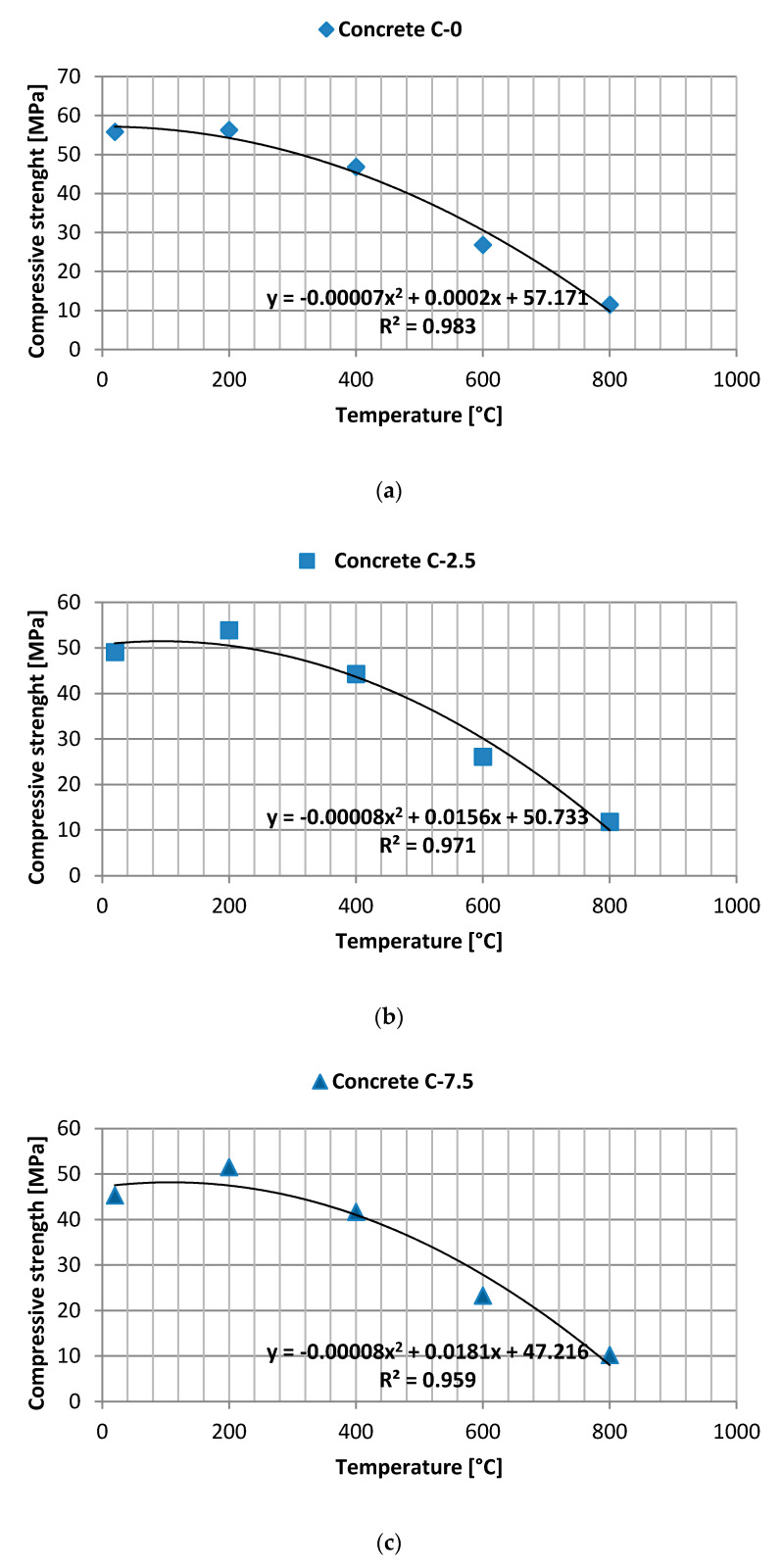
(**a**–**c**) Diagram of negative correlation.

A curve of multiple regression has been plotted for temperatures and for average strength (Figure 8) including a calculation of the correlation equation and application of the growth trend on the dispersion diagram. Once a suitable predictive equation has been obtained in the form of a function of the second degree polynomial, the following questions may be posed: How effective is the prediction for the dependent variable? In addition, what is the accuracy of an assessment of such effectiveness? Table 11 presents relations between two variables, i.e., the r Pearson correlation coefficient (describing direction and force), coefficient of determination R^2^ and the significance level for the given coefficient.

The obtained diagrams present a negative correlation, with a visible dependence ascertained between values, for which a decrease (increase) in strength is accompanied by an increase (decrease) in temperature. The coefficient of determination is significant at the level of 0.001 for C-0, 0.002 for C-2.5 and 0.004 for C-7.5.

### 4.5. Concrete Testing in the Event of a Fire 

**Figure 9:**
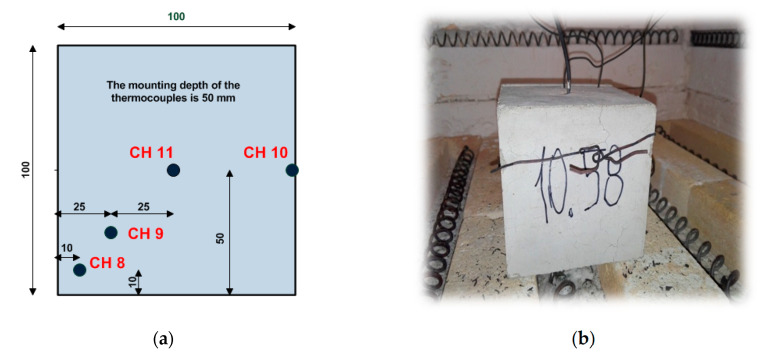
Cubic sample: (**a**) distribution of thermoelements; (**b**) sample with thermocouples.

Before commencing the heating process, all samples were dried to constant mass. The testing was conducted according to the standard temperature-time curve according to EN 1991-1-2:2005 [71]. The samples were heated up in a special furnace at temperatures of 200 °C, 400 °C, 600 °C and 800 °C. The heating time depended on the value of the programmed temperature. The temperature distribution in the pilot sample was recorded with the use of a computer equipped with dedicated software. The temperature distribution was monitored by four thermocouples (Figure 9). Openings for heating thermoelements were made in the central part of the sample (CH 11), 25 mm from the edge of the base (CH 9), 10 mm from the edge of the base (CH 8). The thermocouple (CH 10) was fixed to the lateral surface of the sample. The regulating thermoelement (RT) is introduced through the posterior wall and is close to the top part of the furnace. The depth of the drilling was 50 mm.

**Figure 10:**
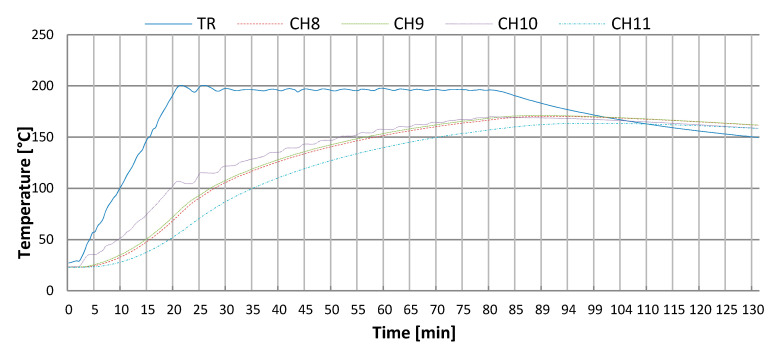
Temperature distribution in pilot sample of C-2.5. Heating curve to 200 °C.

An increase in temperature weakens the structure of the material. Cracking is visible on the sample surface. The actual distribution of temperatures shown in Figure 10 enables observations of the behaviour of concrete in the event of a fire.

Thermocouples distributed in the pilot sample enabled modelling the progress of standardised fire. The thermal process was programmed by specifying the time of heating, temperature, as well as the number of process sections that should be implemented.

The point at which the temperatures become balanced proves that the sample had been heated evenly throughout its entire volume. In the process of cooling down a visible increase occurs in the temperature of the internal layers [5].

### 4.6. Scanning Electron Microscopy (SEM) with Energy Dispersive X-ray Spectroscopy (EDS) and X-ray Diffraction Analysis (XRD)

**Figure 11:**
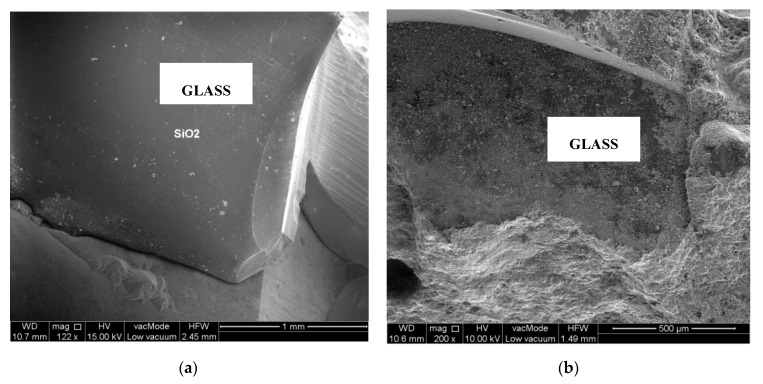
SEM. The microstructure of glass at temperatures of: (**a**) 20 °C; (**b**) 800 °C.

The paper presents a continuation of deliberations commenced in article [5], which are related by a common study subject pertaining to the impact of waste heat resistant glass cullet on the nature of phase transformations that take place in the heating phase. The studies comprised verification of microstructural properties and identification of phase composition. Tests were conducted on fracture surfaces of samples sustained in the destructive tests. Morphology of the glass obtained in the scanning microscope images are shown in Figure 11.

Structural testing of the composition of heat-resistant glass (unheated at 20 °C and heated up to 800 °C) did not detect any significant changes. Glass is characterised by low resistance to the impact of alkali and significant resistance to water and strong acids. Silica glass SiO_2_ is characterised by a relatively low thermal expansion coefficient equalling circa $5\times 10^{-7}K^{-1}$. Consequently, it is resistant to thermal shock and the occurring differences in temperature. 

**Figure 12:**
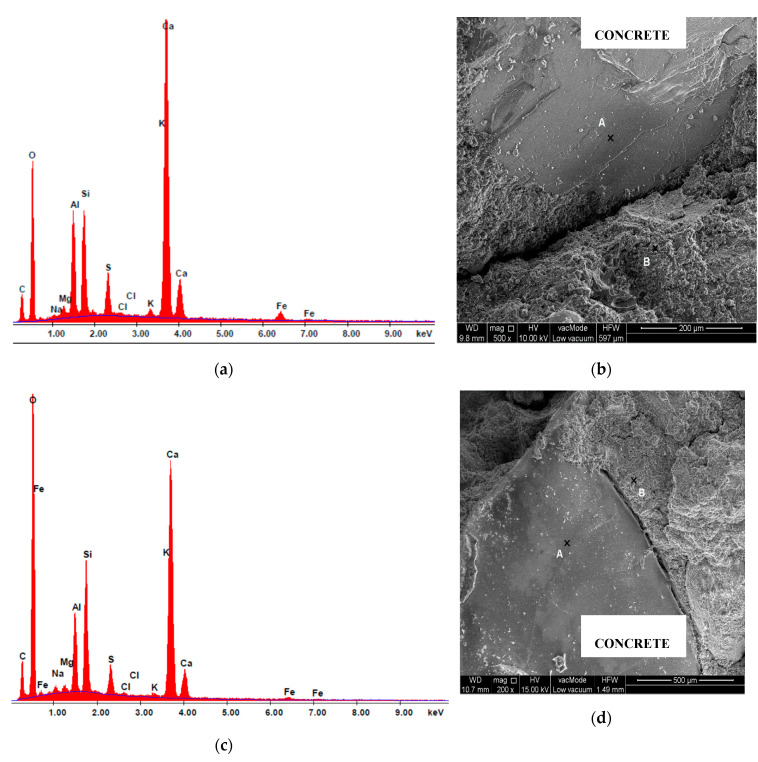
Analysis of the C-7.5 concrete determined from point B: (**a**) chemical composition spectrum of SEM-EDS, 20 °C; (**b**) microstructure, 20 °C; (**c**) chemical composition spectrum of SEM-EDS, 800 °C; (**d**) microstructure, 800 °C.

**Figure 13:**
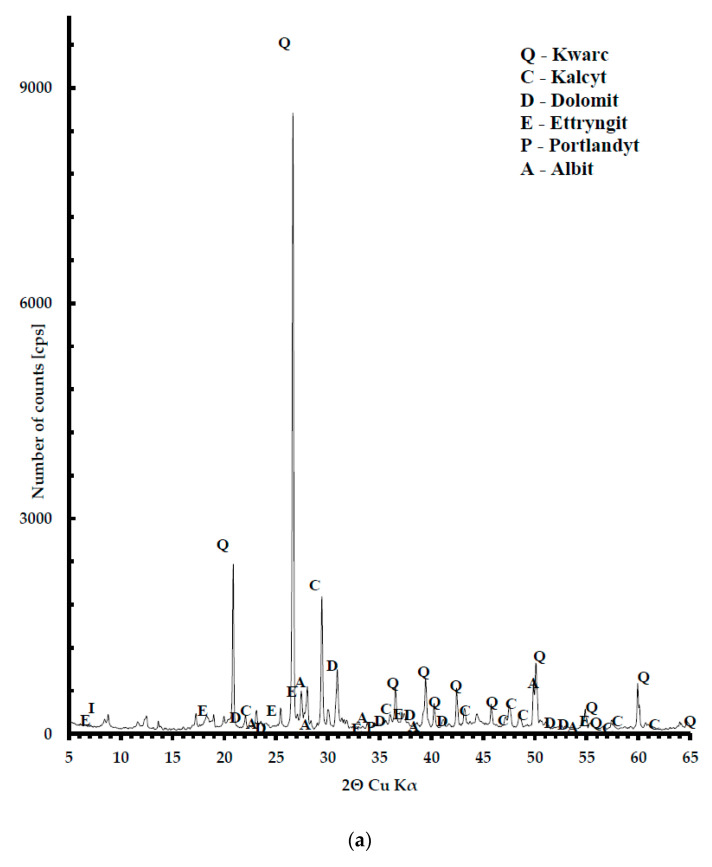

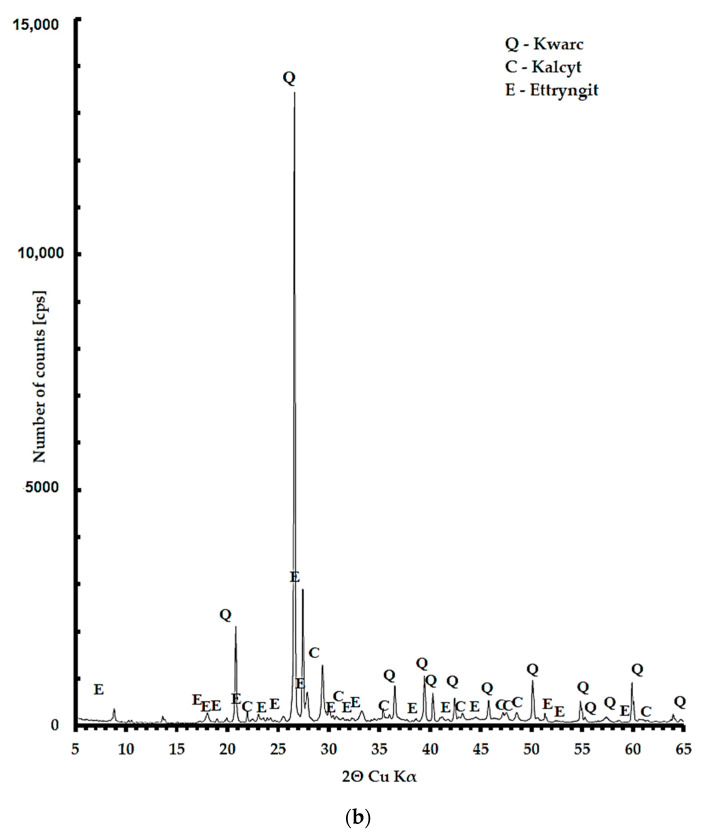
XRD analysis for C-7.5: (**a**) diffractogram, 20 °C; (**b**) diffractogram, 800 °C.

**Figure 14:**
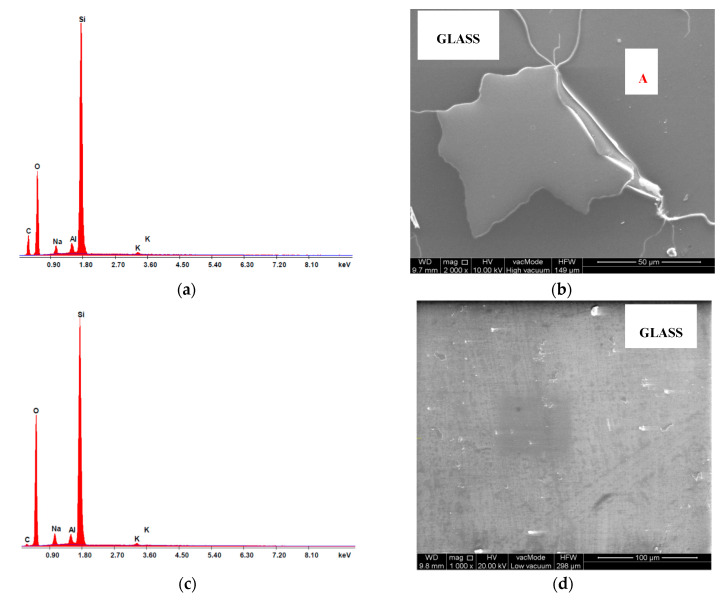
Analysis of glass cullet, 20 °C: (**a**) chemical composition spectrum of SEM-EDS, point A; (**b**) microstructure of glass, point A; (**c**) SEM-EDS chemical composition spectrum, area; (**d**) glass microstructure, area.

The chemical composition in the micro-area of cement composite samples was analysed using the EDS attachment. The testing also comprised analysis of the risk of dehydration of samples described in the literature [5,72]. The oxide composition of glass cullet is presented in Table 12 and Table 13, and selected spectra of the chemical composition are shown in Figure 12, Figure 13 and Figure 14. The chemical bonding of glass has a limited degree of modularity. As a non-crystalline (amorphous) structure, glass is characterised by transition to a liquid state during heating and a high degree of short-range modularity. The X-ray spectrum analysis defines the percentage content of particular oxides. The oxide composition of C-7.5 composite heated at 800 °C, determined from point A, is characterised by high contents of calcium oxide CaO (52.03%), silicon oxide (IV) SiO_2_ (22.32%), alumina Al_2_O_3_ (14.35%), sodium oxide Na_2_O (0.98%) (Table 12).

The phase composition of concrete was determined using X-ray diffraction analysis (Figure 13). It is dominated by quartz recognized by the interplanar distances d = 3.34, 4.25 Å, calcite d = 3.03, 2.85, 2.09 Å and dolomite 2.87, 2.19, 1.78 Å. The sub-components are feldspars in the form of albite d = 3.26, 3.22 and portlandite d = 2.63, 4.90 Å and ettryngit d = 9.76, 3.87 Å. The process of heating to a temperature of 800 °C caused the entire decomposition of dolomite and portlandite, which initiated the process of carbonation of the cement binder.

The glass composition analysis was conducted after glass was subjected to the impact of temperatures of 20 °C and 800 °C, with the determination made from point A and the area of the collected sample. The results are specified in Table 13. An analysis of the chemical composition of recyclate showed slight differences in the content of the main chemical components. The content of silicon oxide increases, at the expense of which the proportion of alkali oxides (Na_2_O and K_2_O) slightly decreases. The content of Al_2_O_3_ ranges from 3.68 to 4.69% which allows the statement that the tested concrete is thermally resistant.

**Figure 15:**
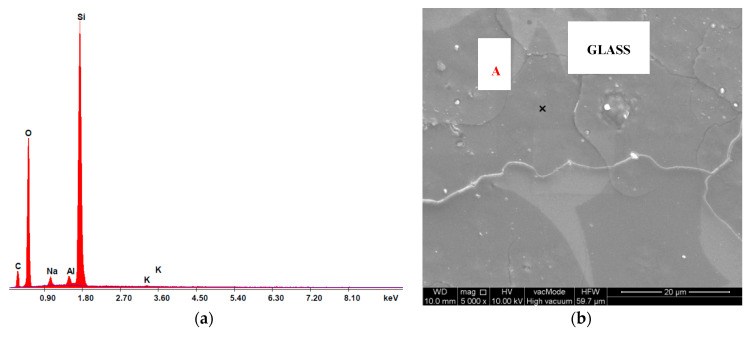

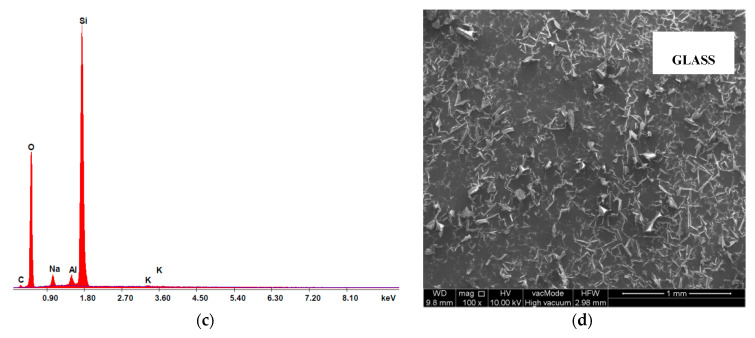
Analysis of oxide composition of glass cullet, 800 °C: (**a**) chemical composition spectrum of SEM-EDS, point A; (**b**) the microstructure of glass, point A; (**c**) SEM-EDS chemical composition spectrum, area; (**d**) glass microstructure, area.

The spectra of the chemical composition (Figure 14a,c and Figure 15a,c) confirm the presence of elements contained by glass cullet that originate from quartz (SiO_2_) and oxides K_2_O, Na_2_O among others. In addition, peaks coming from alumina AL_2_O_3_, which enhanced mechanical, chemical and thermal properties of silicate glass were ascertained.

Based on the conducted structural testing and data obtained from published studies pertaining to analyses of absorption spectra in infrared of Raman spectra, a presumption may be made that cement composite containing an additive of heat-resistant glass is less susceptible to strains resulting from thermal expansion [72,73,74]. The material is less susceptible to cracking after thermal shock.

## 5. Conclusions

Source materials contain limited information as to the impact of fire-resistant cullet on the physical, mechanical and chemical properties of cement composite. Specific data obtained during the conducted experiment and the analysis of obtained results enable the formulation of the following final conclusions:

(1) The average compressive strength of concrete improved using 2.5% of waste glass cullet was found to be 48.6 MPa and 49.1 MPa, respectively, after 28 and 180 days of curing.
(2) Replacement of 2.5% and 7.5% of natural aggregate (gravel and sand) with recycled aggregate allowed the attainment of concrete of class C30/37. Compared to reference concrete, a fall in strength was recorded. It may also be seen that the strength of the composite grows with the extension of the concrete curing time.
(3) The most advantageous temperature of firing amounted to 200 °C. The composite was found to have an increase in strength by 6.2 MPa after 180 days of curing.
(4) An excessively high temperature (800 °C) causes damage to gravel grains which caused an impairment of the properties of hardened concrete. In the composites, compressive strength decreased by circa 50%.
(5) The mass loss of the C-2.5 composite did not exceed 1% (the admissible value is 6%). Concrete may be successfully used as a fire partition wall.
(6) Testing pointed to the existence of concentrations of selected elements, which remained within acceptable levels.
(7) Glass cullet (borosilicate glass) used for testing was not found to have changes to the structure and composition after the impact of temperature at the level of 800 °C.
(8) The correlation between average compressive strength and temperature is significant and equals 0.004 for C-7.5 concrete.

The heat resistant glass cullet is not biodegradable and offers satisfactory performance parameters. Appropriate disposal and processing allow multiple re-use. Based on the conducted studies and analyses it has been proven that waste obtained from broken dishes may serve as perfect supplementation for aggregate of natural origin and can replace coarse aggregate used for concrete. Given environmental standards, it may be presumed that composite is an ecological full-value construction material.

The authors are conducting additional tests to analyse the impact of ground glass cullet on properties of cement composite. Those studies are expected to broaden the available knowledge and to determine features of concrete not included in the programme of the present study.

## Figures and Tables

**Table 1:** Physical and chemical properties of TERMISIL borosilicate glass.

Component	Requirements
Temperature of transformation, °C	535
Temperature of dilatometric softening, °C	635
Lower annealing temperature, °C	520
Upper annealing temperature, °C	550
Permissible working scope, °C	−40 ÷ 300
Density at 20 °C, g/cm^3^	2.23
Hydrolytic resistance of glass grains at temperature of 98 °C	HGB 1
Average coefficient of linear thermal expansion (30 °C; 300 °$C),\;K^{–1}$	$3.55\times 10^{–6}$
SiO_2_, weight %	80
Na_2_O, weight %	4
K_2_O, weight %	1
B_2_O_3_, weight %	13
Al_2_O_3_, weight %	2

**Table 2:** Results of sieve analysis. Glass cullet.

Mesh Size, mm	Residues on a Sieve, g	Sifting, %
63	0.00	100.00
31.5	180.20	93.16
16	1154.50	49.33
8	969.00	12.54
4	243.80	3.28
2	53.00	1.27
1	17.30	0.61
bottom	16.10	0.00

**Table 3:** Physical and mechanical properties of cement CEM I 42.5 R [34].

Start of Binding Time, min	Volume Stability, mm	Proper Surface, cm^2^/g	2-Day Compressive Strength, MPa	28-Day Compressive Strength, MPa
180	0.6	4189	31.1	55.4

**Table 4:** Chemical properties of cement CEM I 42.5 R as (%) [34].

Loss of Ignition	Insoluble Residue	SO_3_	Cl^−^	Na_2_O_eq_
2.85	0.72	3.20	0.07	0.74

**Table 5:** Physical and chemical properties of fly ash [5].

Component	Requirements
Appearance	grey or dark grey powder
Loss of ignition, %	4.5
Chlorines Cl^−^, %	0.1
Anhydride of sulfuric acid SO_3_, %	0.25
Free calcium oxide CaO, %	0.11
Reactive calcium oxide CaO, %	1.8
Grain density, kg/m^3^	2050
Volume weakness, mm	1
Fineness, %	30
Index of pozzolanic activity after 28 days, %	80
Index of pozzolanic activity after 90 days, %	88
Commencement of binding time as compared to reference concrete of CEM I 42.5R, min	285

**Table 6:** Physical and chemical properties of the Master Pozzolith 18BVC.

Component	Requirements
Basic raw material	lignin sulphonates
Form	liquid
Colour	dark brown
Density (at 20 °C)	1.10 ± 0.02 g/cm^3^
pH (at 20 °C)	5.2 ± 1.0
Contents of chlorides	≤0.1% of mass
Contents of alkali	≤3.0% of mass

**Table 7:** Weight and volume composition of source mixture.

Concrete Ingredients	Concrete		
	C-0	C-2.5	C-7.5
Cement CEM I 42.5 R, kg/m^3^	260	260	260
Fly ash, kg/m^3^	100	100	100
Sand 0/2 mm, kg/m^3^	727	727	727
Gravel 2/16 mm, kg/m^3^	1076	1049	996
Glass 0/16 mm, kg/m^3^	0	27	80
Superplasticiser, kg/m^3^	1.82	1.82	1.82
Water, kg/m^3^	160	160	160
w/c	0.62	0.62	0.62
Sand point, %	46.7	48.7	51.12
Consistence class	S2/S3	S3/S4	S3/S4

**Table 8:** Apparent density of concrete.

Concrete	C-0	C-2.5	C-7.5
Measurement 1	2378	2334	2287
Measurement 2	2391	2363	2345
Measurement 3	2391	2330	2350
Average density, kg/m^3^	2386.7	2342.3	2327.3
Standard deviation from test, kg/m^3^	7.51	18.01	35.02
Standard error, kg/m^3^	4.33	10.40	20.22
Variability coefficient, %	0.31	0.77	1.51

**Table 9:** Elementary statistics for water absorption.

Concrete	C-0	C-2.5	C-7.5
Average absorbability, %	3.7	5.1	4.8
Standard deviation from test, %	0.18	0.26	0.25
Standard error, %	0.05	0.07	0.06
Variability coefficient, %	4.77	5.02	5.10

**Table 10:** Basic statistical parameters. Features of concrete.

Concrete	C-0	C-2.5	C-7.5
**After 28 days**			
Average compressive strength, MPa	53.8	48.6	43.9
Standard deviation in test, MPa	0.65	0.67	1.19
Standard error, MPa	0.38	0.38	0.69
Variability coefficient, %	1.21	1.37	2.72
**After 180 days**			
Average compressive strength, MPa	55.8	49.1	45.3
Standard deviation in test MPa	1.90	1.05	0.25
Standard error, MPa	1.10	0.61	0.15
Variability coefficient, %	3.41	2.14	0.56

**Table 11:** Specification of relations between variables temperature—average strength.

Concrete	C-0	C-2.5	C-7.5
r Pearson correlation coefficient	−0.991	−0.985	−0.979
Coefficient of determination R^2^	0.983	0.971	0.959
Significance level α	0.001	0.002	0.004

**Table 12:** Oxide composition of C-7.5 concrete with addition of heat-resistant glass determined from point A, weight %.

Oxides	20°C	800°C
	Point A	
Na_2_O	0.71%	0.98%
MgO	1.65%	0.88%
Al_2_O_3_	19.10%	14.35%
SiO_2_	17.60%	22.32%
SO_3_	7.99%	6.49%
Cl_2_O	0.30%	0.36%
K_2_O	0.82%	0.56%
CaO	48.17%	52.03%
Fe_2_O_3_	3.64%	2.02%
Total	100.00%	100.00%

**Table 13:** Analysis of composition of glass cullet determined from point A and from the area—weight %.

Oxides	20 °C	800 °C	20 °C	800 °C
	Point A		Area	
Na_2_O	2.81%	2.76%	3.99%	3.38%
Al_2_O_3_	4.69%	3.68%	4.61%	3.92%
SiO_2_	91.85%	93.29%	90.72%	92.32%
K_2_O	0.62%	0.26%	0.68%	0.37%
SUMA	100.00%	100.00%	100.00%	100.00%
